# Association between anti-thyroid peroxidase antibody and thyroid stimulating hormone: a cross-sectional study

**DOI:** 10.1038/s41598-023-40275-6

**Published:** 2023-09-01

**Authors:** Yuji Shimizu, Mutsumi Matsuyama, Yuko Noguchi, Midori Takada, Shin-Ya Kawashiri, Shoichi Fukui, Seiko Nakamichi, Yasuhiro Nagata, Takahiro Maeda, Naomi Hayashida

**Affiliations:** 1 Department of General Medicine, Nagasaki University Graduate School of Biomedical Sciences, 1-7-1 Sakamoto Nagasaki-shi, Nagsaki, 852-8523 Japan; 2 Epiedmiology Section, Division of Public Health, Osaka Institute of Public Health, Osaka, 537-0025 Japan; 3 Division of Strategic Collaborative Research, Atomic Bomb Disease Institute, Nagasaki University, Nagasaki, 852-8523 Japan; 4 Department of Community Medicine, Nagasaki University Graduate School of Biomedical Sciences, Nagasaki, 852-8523 Japan; 5 Leading Medical Research Core Unit, Nagasaki University Graduate School of Biomedical Sciences, Nagasaki, 852-8523, Japan; 6 Nagasaki University Health Center, Nagasaki, 852-8521, Japan

**Keywords:** Diagnostic markers, Thyroid diseases, Risk factors

## Abstract

To maintain normal level of thyroid hormone, especially for free thyroxine (FT4), individuals with latent thyroid gland damage might have required higher thyroid stimulating hormone (TSH) than those without latent thyroid gland damage. Anti-thyroid peroxidase antibody (TPO-Ab) is a main cause of auto-immune thyroiditis, and therefore euthyroid individuals positive for TPO-Ab might have latent damage to the thyroid gland. Therefore, the association between TSH values and TPO-Ab positivity may be useful to determine the influence of latent thyroid gland damage on requirement of TSH. Furthermore, because latent damage of thyroid might elevate TSH level but not FT4 level, those associations should be observed independent from FT4. This cross-sectional study analyzed 1431 Japanese with normal ranges of free triiodothyronine (FT3) and FT4. Since TPO-Ab is associated with atherosclerosis in euthyroid individuals, cardiovascular risk factors might underlie the association between TPO-Ab and TSH values. After adjusting for FT4 and known cardiovascular risk factors, the adjusted odds ratio (95% confidence interval) of TPO-Ab positivity for logarithmic value of TSH was 1.53 (1.20, 1.95). Essentially the same association was observed when the analysis was restricted to individuals without subclinical hypothyroidism (1.54 [1.15, 2.13]). Euthyroid individuals with latent thyroid gland damage might have increased the requirement of TSH.

## Introduction

Autoimmune thyroiditis is the most common reason for subclinical hypothyroidism (SCH), defined as an elevated serum thyroid-stimulating hormone (TSH) level with a normal level of free thyroxine (FT4)^1^. Anti-thyroid peroxidase antibody (TPO-Ab) is a known cause of autoimmune thyroiditis.

However, even when TSH levels are within the normal range, they have been shown to correlate with TPO-Ab titers^2^. Therefore, in individuals with TPO-Ab positivity, including those who are euthyroid, maintaining normal levels of thyroid hormones, specifically free triiodothyronine (FT3) and FT4, requires higher TSH levels than in people who are TPO-Ab negative because latent thyroid gland damage causes lower production of these hormones.

To ensure normal levels of these hormones, the requirement of TSH for thyroid gland is elevated among those with latent thyroid gland damage, independent from FT4, TPO-Ab-positive individuals must be required higher TSH levels than TPO-Ab-negative individuals. Furthermore, elevates the requirement of TSH might be observed even among those of TPO-Ab-positive individuals within normal range of TSH. Those participants who have normal range of thyroid hormone but elevates the level of TSH regraded as having subclinical hypothyroidism (SCH). Therefore, the association between TPO-Ab positive and TSH could be observed among those without SCH.

Other previous studies reported significant association between TPO-Ab and atherosclerosis in euthyroid individuals^3,4^. Since atherosclerosis is established cardiovascular risk factors, cardiovascular risk factors also could act as confounder on the association between TOP-Ab-positive and TSH levels.

Therefore, we hypothesized that independent from FT4 and known cardiovascular risk factors, serum TSH would be significantly positively associated with TPO-Ab positivity among individuals with normal thyroid hormone levels. We also expected the same association to be present when the analysis was limited to individuals without SCH.

To investigate our hypothesis, we conducted a cross-sectional study in 1,431 individuals within normal ranges of thyroid hormones (FT3 and FT4) who underwent an annual health check-up in 2014.

## Material and methods

### Study population

This study was approved by the Ethics Committee of Nagasaki University Graduate School of Biomedical Sciences (project registration number 14051404-13). Informed consent was obtained from all participants. Written consent forms were used to ensure that participants under stood the objectives of the study when obtaining informed consent. All procedures involving human participants were performed in accordance with the ethical standards of the institution research committee and with the 1964 Helsinki Declaration and its later amendments for comparable ethical standards.

This cross-sectional study enrolled 1883 Japanese individuals aged 40–74 years from the town of Saza in western Japan. These individuals underwent an annual medical check-up in 2014 as recommended by the Japanese government.

To avoid the influence of thyroid disease, individuals were excluded if they met any of the following criteria: had a history of thyroid disease (n = 60); had missing thyroid function data, such as levels of TSH, FT3, or FT4 (n = 17); or had FT3 and/or FT4 levels outside the normal range (n = 77). In addition, participants without TPO-Ab data (n = 295) were exeluded. Individuals without smoking and drinking status data (n = 2) or BMI data (n = 1) were also excluded. A total of 1,431 participants with a mean age of 60.8 years (standard deviation (SD): 9.0 years; range: 40–74 years) were included in the study.

### Data collection and laboratory measurements

Trained interviewers obtained information on clinical characteristics. A fasting blood sample was collected from each participant. TSH, FT3, and FT4 levels were measured with chemiluminescent immunoassays by the LSI Medience Corporation (Tokyo, Japan). The normal range for FT3 (2.1–4.1 pg/mL), FT4 (1.0–1.7 ng/dL), and TSH (0.39–4.01 μIU/mL) based on this method are described elsewhere^5^. TPO-Ab titers were measured using standard procedures by the LSI Medience Corporation; the normal range (negative) was < 16 IU/mL^5^. Hemoglobin A1c (HbA1c), triglycerides, and high-density lipoprotein cholesterol (HDLc) were also measured using standard procedures by SRL, Inc. (Tokyo, Japan). Individuals with a TSH serum concentration > 4.01 μIU/mL were defined as having SCH. Individuals with a TPO-Ab titer ≥ 16 IU/mL were defined as TPO-Ab positive.

### Statistical analysis

The clinical characteristics of the study population were compared by TSH quartiles. Quartile values of TSH for men were < 1.08 [μIU/mL] for Q1 (low), 1.08–1.53 [μIU/mL] for Q2, 1.54–2.17 [μIU/mL] for Q3, and 2.18 ≤ [ng/mL] for Q4 (high). The corresponding values for women were < 1.13 [ng/mL], 1.13–1.61 [ng/mL], 1.62–2.32 [ng/mL], and 2.33 ≤ [ng/mL]. Data are presented as means ± SD or n (%), except for triglycerides. Since triglycerides values had a skewed distribution, they were expressed based on the median (interquartile range). Trend tests were also performed.

Logistic regression models were used to calculate odds ratios (ORs) and 95% confidence intervals (95% CIs) to determine the association between TSH values and TPO-Ab positivity.

Adjustment for confounding factors was performed in four ways. Model 1 adjusted only for sex and age. Model 2 adjusted for sex and age, plus FT4 level. Model 3 adjusted for sex, age, and FT4 plus thyroid hormone activity as measured by FT3 level. Since the presence of TPO-Ab was associated with atherosclerosis in euthyroid individuals^3,4^, and subclinical hypothyroidism was reported to be associated with atherosclerosis^6^, cardiovascular risk factors might confound the association between TPO-Ab and TSH values. Model 4 adjusted for sex, age, and FT4 as well as for known cardiovascular risk factors, as follows: systolic blood pressure (SBP), body mass index (BMI), drinking status (non, often, daily), smoking status (never, former, current), triglycerides, HDLc, and HbA1c.

The Hosmer–Lemeshow test was used to assess goodness of fit in all logistic regression analyses, and it validated the use of the present study population in each analysis.

All statistical analyses were performed with SAS for Windows (version 9.4; SAS Inc., Cary, NC). Values of p < 0.05 were regarded as statistically significant.

## Results

Of the 1431 participants in this study, 266 were diagnosed as TPO-Ab positive and 81 were diagnosed with SCH.

### Cardiovascular risk factors according to TSH quartiles

Table 1 shows cardiovascular risk characteristics of the study population by TSH quartiles. TSH quartiles were significantly positively associated with age, BMI, triglycerides and HbA1c, and inversely associated with current smoking, HDLc, FT3, and FT4.

**Table 1:** Characteristics (known cardiovascular risk factors) of study population by levels of thyroid stimulating hormone (TSH).

	TSH (quartiles)				*p*
	Q1 (low)	Q2	Q3	Q4 (high)	
No of participants	359	359	356	357	
Men, %	37.0	37.0	37.4	37.0	1.000
Age, year	60.4 ± 9.2	60.3 ± 8.9	60.4 ± 9.1	62.3 ± 8.8	0.006
SBP, mmHg	124 ± 18	124 ± 16	125 ± 17	127 ± 17	0.110
BMI, kg/m^2^	22.4 ± 3.2	22.7 ± 3.3	23.0 ± 3.4	23.3 ± 3.6	0.003
Daily drinker, %	43.7	42.6	38.8	35.0	0.071
Non-drinker, %	54.3	54.3	57.9	59.7	0.375
Current smoker, %	20.9	16.7	13.8	9.0	< 0.001
Former smoker, %	22.3	19.5	19.4	19.9	0.744
Triglycerides. mg/dL	83 [62,113]*^1^	87 [65,119]*^1^	88[66,125]*^1^	97[68,136]*^1^	0.013*^2^
HDL-cholesterol, mg/dL	61 ± 15	61 ± 15	61 ± 15	59 ± 14	0.026
HbA1c, %	5.6 ± 0.5	5.6 ± 0.6	5.7 ± 0.7	5.7 ± 0.7	0.007
FT3, (2.1–4.1) pg/mL	3.2 ± 0.3	3.2 ± 0.3	3.2 ± 0.3	3.1 ± 0.3	0.004
FT4, (1.0–1.7) ng/dL	1.3 ± 0.2	1.3 ± 0.2	1.2 ± 0.2	1.2 ± 0.2	< 0.001

TSH, thyroid stimulating hormone. SBP, systolic blood pressure; BMI, body mass index; HDL, high density lipoprotein; HbA1c, hemoglobin A1c. FT3; free triiodothyronine; FT4, free thyroxine; Values are mean ± standard deviation. *1: Values are median [the first quartile, third quartile]. *2: Logarithmic transformation was used. Quartile values of TSH for men were < 1.08 [μIU/mL] for Q1 (low), 1.08–1.53 [μIU/mL] for Q2, 1.54–2.17 [μIU/mL] for Q3, and 2.18 1 ≤ [μIU/mL] for Q4 (high). The corresponding values for women were < 1.13 [μIU/mL], 1.13–1.61 [μIU/mL], 1.62–2.32 [μIU/mL], and 2.33 ≤ [μIU/mL].

### TPO-Ab positivity according to TSH quartiles

Table 2 shows the association between TSH quartiles and TPO-Ab positivity. TSH quartiles were significantly positively associated with TPO-Ab positivity. This association remained even after adjustment for FT4 (Model 2), FT3 and FT4 (Model 3), FT4 and known cardiovascular risk factors (Model 4).

**Table 2:** Association between anti-thyroid peroxidase antibody (TPO-Ab) positive and thyroid stimulating hormone (TSH).

	TSH (quartiles)				p	TSH (logarithmic)
	Q1 (low)	Q2	Q3	Q4 (high)		
Total						
No of participants	359	359	356	357		
No of case (%)	51 (14.2)	53 (14.8)	72 (20.2)	90 (25.2)		
Model 1	Ref	1.05 (0.69, 1.59)	1.28 (0.85, 1.92)	1.99 (1.36, 2.91)	< 0.001	1.51 (1.19, 1.90)
Model 2	Ref	1.07 (0.70, 1.62)	1.58 (1.06, 2.34)	2.09 (1.42, 3.08	< 0.001	1.57 (1.24, 2.00)
Model 3	Ref	1.07 (0.70, 1.62)	1.57 (1.06, 2.33)	2.08 (1.41, 3.05)	< 0.001	1.56 (1.23, 1.98)
Model 4	Ref	1.07 (0.70, 1.62)	1.55 (1.04, 2.31)	2.04 (1.38, 3.02)	< 0.001	1.53 (1.20, 1.95)

Ref: reference. Model 1: adjusted for sex and age. Model 2: (Model 1 +) further adjusted for free thyroxine (FT4). Model 3: (Model 2 +) further adjusted for free triiodothyronine (FT3). Model 4: (Model 2 +) further adjusted for systolic blood pressure, body mass index, drinking status, smoking status, triglyceride, high density lipoprotein cholesterol (HDLc), and hemoglobin A1c (HbA1c).

### Sex-specific association between TPO-Ab positivity and TSH quartiles

Sex-specific analysis revealed essentially the same associations between men and women (Table 3).

**Table 3:** Sex-specific association between anti-thyroid peroxidase antibody (TPO-Ab) positive and thyroid stimulating hormone (TSH).

	TSH (quartiles)				p	TSH (logarithmic)
	Q1 (low)	Q2	Q3	Q4 (high)		
Men						
No of participants	133	133	133	132		
No of case (%)	18 (13.5)	16 (12.0)	21 (15.8)	35 (26.5)		
Model 1	Ref	0.87 (0.42, 1.79)	1.20 (0.61, 2.38)	2.24 (1.19, 4.22)	0.005	1.54 (1.05, 2.26)
Model 2	Ref	0.90 (0.44, 1.87)	1.25 (0.63, 2.43)	2.36 (1.24, 4.50)	0.004	1.59 (1.07, 2.36)
Model 3	Ref	0.88 (0.43, 1.82)	1.22 (0.61, 2.42)	2.29 (1.20, 4.34)	0.005	1.56 (1.05, 2.31)
Model 4	Ref	0.90 (0.43, 1.87)	1.31 (0.65, 2.63)	2.45 (1.27, 4.75)	0.003	1.61 (1.07, 2.43)
Women						
No of participants	226	226	223	225		
No of case (%)	33 (14.6)	37 (16.4)	51 (22.9)	55 (24.4)		
Model 1	Ref	1.15 (0.69, 1.92)	1.74 (1.07, 2.81)	1.85 (1.15, 2.99)	0.003	1.50 (1.11, 2.01)
Model 2	Ref	1.16 (0.70, 1.93)	1.78 (1.09, 2.89)	1.97 (1.21, 3.19)	0.002	1.57 (1.16, 2.13)
Model 3	Ref	1.16 (0.70, 1.94)	1.78 (1.09, 2.89)	1.96 (1.21, 3.18)	0.002	1.57 (1.16, 2.12)
Model 4	Ref	1.12 (0.67, 1.88)	1.66 (1.02, 2.72)	1.81 (1.10, 2.96)	0.006	1.49 (1.10, 2.02)

Ref: reference. Model 1: adjusted for sex and age. Model 2: (Model 1 +) further adjusted for free thyroxine (FT4). Model 3: (Model 2 +) further adjusted for free triiodothyronine (FT3). Model 4: (Model 2 +) further adjusted for systolic blood pressure, body mass index, drinking status, smoking status, triglycerides, high density lipoprotein cholesterol (HDLc), and hemoglobin A1c (HbA1c).

### Association between TPO-Ab positivity and TSH quartiles among participants without SCH

Table 4 shows the association between TPO-Ab positivity and TSH quartiles among participants without SCH. TPO-Ab positivity was significantly positively associated with TSH quartiles independently of FT3 and known cardiovascular risk factors.

**Table 4:** Association between anti-thyroid peroxidase antibody (TPO-Ab) positive and thyroid stimulating hormone (TSH) among participants without subclinical hypothyroidism (SCH).

	TSH (quartiles)				p	TSH (logarithmic)
	Q1 (low)	Q2	Q3	Q4 (high)		
Total						
No of participants	359	359	356	276		
No of case (%)	51 (14.2)	53 (14.8)	72 (20.2)	69 (25.0)		
Model 1	Ref	1.05 (0.69, 1.59)	1.53 (1.04, 2.28)	1.96 (1.31, 2.94)	< 0.001	1.53 (1.14, 2.04)
Model 2	Ref	1.07 (0.71, 1.63)	1.58 (1.07, 2.35)	2.06 (1.37, 3.10)	< 0.001	1.59 (1.18, 2.13)
Model 3	Ref	1.07 (0.71, 1.63)	1.58 (1.06, 2.34)	2.05 (1.37, 3.08)	< 0.001	1.58 (1.18, 2.12)
Model 4	Ref	1.07 (0.70, 1.62)	1.55 (1.04, 2.31)	2.01 (1.33, 3.04)	< 0.001	1.54 (1.15, 2.13)

Ref: reference. Model 1: adjusted for sex and age. Model 2: (Model 1 +) further adjusted for free thyroxine (FT4). Model 3: (Model 2 +) further adjusted for free triiodothyronine (FT3). Model 4: (Model 2 +) further adjusted for systolic blood pressure, body mass index, drinking status, smoking status, triglycerides, high density lipoprotein cholesterol (HDLc), and hemoglobin A1c (HbA1c).

### Sex-specific association between TPO-Ab positivity and TSH quartiles among participants without SCH

Table 5 shows the sex-specific association between TPO-Ab positivity and TSH quartiles among participants without SCH. In both men and women, TPO-Ab positivity was significantly positively associated with TSH quartiles independently of FT4.

**Table 5:** Sex-specific association between anti-thyroid peroxidase antibody (TPO-Ab) positive and thyroid stimulating hormone (TSH) among participants without subclinical hypothyroidism (SCH).

	TSH (quartiles)				p	TSH (logarithmic)
	Q1 (low)	Q2	Q3	Q4 (high)		
Men						
No of participants	133	133	133	99		
No of case (%)	18 (13.5)	16 (12.0)	21 (15.8)	29 (29.3)		
Model 1	Ref	0.87 (0.42, 1.79)	1.20 (0.61, 2.38)	2.56 (1.32, 4.97)	0.003	1.88 (1.12, 3.14)
Model 2	Ref	0.92 (0.44, 1.91)	1.27 (0.64, 2.53)	2.76 (1.41, 5.42)	0.002	2.03 (1.20, 3.43)
Model 3	Ref	0.90 (0.43, 1.86)	1.24 (0.62, 2.46)	2.66 (1.36, 5.19)	0.003	1.94 (1.15, 3.26)
Model 4	Ref	0.91 (0.44, 1.89)	1.31 (0.65, 2.63)	2.76 (1.41, 5.42)	0.002	1.98 (1.16, 3.38)
Women						
No of participants	226	226	223	170		
No of case (%)	33 (14.6)	37 (16.4)	51 (22.9)	40 (23.5)		
Model 1	Ref	1.15 (0.69, 1.92)	1.73 (1.07, 2.81)	1.68 (1.01, 2.80)	0.014	1.38 (0.97, 1.96)
Model 2	Ref	1.16 (0.70, 1.94)	1.78 (1.09, 2.89)	1.76 (1.05, 2.95)	0.009	1.43 (1.01, 2.05)
Model 3	Ref	1.16 (0.70, 1.94)	1.78 (1.09, 2.89)	1.76 (1.05, 2.94)	0.009	1.43 (1.01, 2.04)
Model 4	Ref	1.11 (0.66, 1.86)	1.66 (1.02, 2.72)	1.61 (0.95, 2.72)	0.025	1.35 (0.94, 1.93)

Ref: reference. Model 1: adjusted for age. Model 2: (Model 1 +) further adjusted for free thyroxine (FT4). Model 3: (Model 2 +) further adjusted for free triiodothyronine (FT3). Model 4: (Model 2 +) further adjusted for systolic blood pressure, body mass index, drinking status, smoking status, triglycerides, high density lipoprotein cholesterol (HDLc), and hemoglobin A1c (HbA1c).

In model 4, for women, even the statistical power could not reach significant value, essentially same association was observed with men; positive tendency between TSH and TPO-Ab positively was observed.

## Discussion

The main finding of this study was that among euthyroid individuals, independent of FT4 and known cardiovascular risk factors, TSH values within the normal range were positively associated with TPO-Ab positivity. TSH is a hormone that stimulates the production of thyroid hormones by the thyroid gland.

A negative feedback system exists in which high levels of thyroid hormones such as FT4 inhibit the production of TSH by the pituitary gland, and therefore participants with lower levels of FT4 elevates the TSH level. In such cases, elevated TSH stimulates the production of thyroid hormone which increase FT4. By this using system, thyroid hormone levels are preciously regulated.

Generally, elevated serum TSH levels with normal FT4 levels are regarded as SCH. Since positive association between blood pressure and TSH within normal range is reported^7^, even among within normal range of TSH, TSH could indicates the thyroid function. To maintain FT4 within a normal range, greater TSH stimulation is required in individuals with latent thyroid gland damage than in those without. Given the feedback system that regulates these hormones, TSH production should be stronger for individuals with such damage than those without.

Decreased sensitivity of the thyroid gland to TSH is associated with higher TSH values, therefore, a decrease in this sensitivity should be associated with latent thyroid damage. These associations should be independent from FT4, because in our present hypothesis, not FT4 level itself but latent damage of thyroid might determine the serum concentration of TSH.

Because the presence of TPO-Ab might induce latent thyroid gland damage among individuals with normal thyroid hormone levels, TSH values could be significantly positively associated with TPO-Ab positivity, as shown in the present study. This positive association was observed in this study even when we limited the analysis to individuals within normal range of TSH.

Since thyroid cysts have been shown to be inversely associated with TPO-Ab titers among individuals with normal thyroid hormone levels^8^, the absence of such cysts may partly indicate the presence of latent thyroid gland damage. Thyroid cysts could have clinical implications because they support thyroid activity^9^. In our previous study in a general population of 1724 individuals, the correlation between TSH and thyroid hormones (FT3 and FT4) was slightly stronger in participants without thyroid cysts than those with them; the simple correlations (r) of TSH with FT3 and FT4, respectively, were r = − 0.13 (p < 0.001) and r = − 0.18 (p < 0.001) in those without thyroid cysts, and r = − 0.03 (p = 0.525) and r = − 0.09 (p = 0.030) in those with thyroid cysts^10^. These results indicate that even among participants with normal ranges of thyroid hormones (FT3, FT4), the sensitivity of these hormones to TSH secretion could be slightly stronger in those with latent thyroid gland damage than in those without.

This is the first study to report a positive association between a normal range of TSH values and TPO-Ab positivity in euthyroid individuals independent from FT4 and known cardiovascular risk factors. This association indicates that TPO-Ab positively might elevates TSH level by reducing the effectiveness of production of thyroid hormone known as FT4 in latent damaged thyroid gland.

This study has important clinical implications. Even among euthyroid individuals, the presence of TPO-Ab might injure the thyroid gland. Further investigation is necessary to determine if TSH values in the normal range can be used to evaluate latent thyroid gland damage.

Potential limitations of the present study warrant consideration. Due to the limited number of blood samples, we could not evaluate the influence of anti-thyroglobulin antibodies, which may act as a strong confounding factor. It is necessary to perform further investigations using these data. Moreover, this was a cross-sectional study and therefore no causal relationships could be established.

## Conclusion

In conclusion, independent from FT4 and known cardiovascular risk factors, TSH values within the normal range were positively associated with TPO-Ab positivity in a euthyroid population. While further investigation is necessary, this finding indicates that the TSH may be a useful tool to clarify the influence of TPO-Ab on latent thyroid gland damage.

## Data Availability

We cannot publicly provide individual data due to participant privacy, according to ethical guidelines in Japan. Additionally, the informed consent was obtained does not include a provision for publicity sharing data. Qualifying researchers may apply to access a minimal dataset by contacting Prof Naomi Hayashida, Principal Investigator, Division of Promotion of Collaborative Research on Radiation and Environment Health Effects, Atomic Bomb Disease Institute, Nagasaki University, Nagasaki, Japan at naomin@nagasaki-u.ac.jp. Or, please contact the office of data management at ritouken@vc.fctv-net.jp. Information for where data request is also available at https://www.genken.nagasaki-u.ac.jp/dscr/message/ and http://www.med.nagasaki-u.ac.jp/cm/.
